# Bis(6′-carb­oxy-2,2′-bipyridine-6-carboxyl­ato-κ^3^ *N*,*N*′,*O* ^6^)nickel(II) tetra­hydrate

**DOI:** 10.1107/S1600536809006515

**Published:** 2009-02-28

**Authors:** Huimin Wang, Haiquan Su, Jinjin Xu, Fenghua Bai, Ya Gao

**Affiliations:** a School of Chemistry and Chemical Engineering, Inner Mongolia University, Hohhot 010021, People’s Republic of China

## Abstract

In the title compound, [Ni(C_12_H_7_N_2_O_4_)_2_]·4H_2_O, the Ni atom is located at the centre of a distorted octa­hedron, formed by four N atoms and two O atoms from the same two tridentating chelated 6-carb­oxy-2,2′-bipyridine-6′-carboxyl­ate (*L*) ligands. Face-to-face π-stacking inter­actions between inversion-related pyridine rings with centroid–centroid distances of 3.548 (3) and 3.662 (3) Å (perpendicular distances between the respective rings are 3.314 and 3.438 Å) are found. Inter­molecular O—H⋯O hydrogen bonds between water mol­ecules and *L* ligands form *R*_5_^3^(10), *R*_6_^5^(14) and *R*_5_^5^(12) rings and also a centrosymmetric cage-like unit of water mol­ecules, which link eight adjacent Ni^II^ centers, forming a three-dimensional framework.

## Related literature

For hydrogen-bonding motifs, see: Bernstein *et al.* (1995 ▶). For the structural and photophysical properties of Ln^III^ complexes with the title ligand, see: Bünzli *et al.* (2000 ▶). For a *catena*-poly diaqua Cd^II^ complex with the title ligand, see: Knight *et al.* (2006 ▶). For an explanation of ‘ligand star’, see: Gao *et al.* (2006 ▶). For the structural characterization and fluorescent properties of Ln^III^ complexes with pyridine-2,6-dicarboxylic acid, see: Liu *et al.* (2008 ▶). For the structural and photophysical properties of Eu^III^ complexes with 2,2′-dipyridine-4, 4′-dicarboxylic acid, see: Law *et al.* (2007 ▶). For the structural properties of a metal-organic framework (MOF) based on Pt, Y and 2,2′-bipyridine-5,5′-dicarboxyl­ate, see: Szeto *et al.* (2006 ▶). For a review of the properties of coordination polymer networks *via* O– and N-atoms, see: Robin & Fromm (2006 ▶).
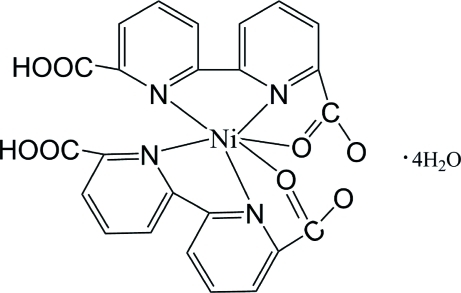


## Experimental

### 

#### Crystal data

[Ni(C_12_H_7_N_2_O_4_)_2_]·4H_2_O
*M* *_r_* = 617.17
Triclinic, 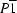

*a* = 9.990 (2) Å
*b* = 10.896 (2) Å
*c* = 12.565 (3) Å
α = 112.97 (3)°
β = 100.09 (3)°
γ = 90.30 (3)°
*V* = 1235.7 (4) Å^3^
*Z* = 2
Mo *K*α radiation
μ = 0.86 mm^−1^
*T* = 113 K
0.20 × 0.10 × 0.08 mm

#### Data collection

Rigaku Saturn CCD area-detector diffractometer
Absorption correction: multi-scan (*CrystalClear*; Rigaku/MSC, 2005 ▶) *T*_min_ = 0.846, *T*_max_ = 0.934
7165 measured reflections
4305 independent reflections
2745 reflections with *I* > 2σ(*I*)
*R*_int_ = 0.126

#### Refinement

*R*[*F* ^2^ > 2σ(*F* ^2^)] = 0.068
*wR*(*F* ^2^) = 0.211
*S* = 1.05
4305 reflections
372 parameters
H-atom parameters constrained
Δρ_max_ = 0.96 e Å^−3^
Δρ_min_ = −0.75 e Å^−3^

### 

Data collection: *CrystalClear* (Rigaku/MSC, 2005 ▶); cell refinement: *CrystalClear*; data reduction: *CrystalClear*; program(s) used to solve structure: *SHELXS97* (Sheldrick, 2008 ▶); program(s) used to refine structure: *SHELXL97* (Sheldrick, 2008 ▶); molecular graphics: *DIAMOND* (Brandenburg & Putz, 2006 ▶) and *Mercury* (Macrae *et al.*, 2006 ▶); software used to prepare material for publication: *publCIF* (Westrip, 2009 ▶).

## Supplementary Material

Crystal structure: contains datablocks I, global. DOI: 10.1107/S1600536809006515/si2152sup1.cif

Structure factors: contains datablocks I. DOI: 10.1107/S1600536809006515/si2152Isup2.hkl

Additional supplementary materials:  crystallographic information; 3D view; checkCIF report

## Figures and Tables

**Table:** 

Ni1—N1	1.975 (4)
Ni1—N3	1.987 (4)
Ni1—O1	2.104 (4)
Ni1—O5	2.136 (4)
Ni1—N2	2.161 (5)
Ni1—N4	2.197 (4)

**Table:** 

N3—Ni1—N2	111.50 (16)
N1—Ni1—N4	103.32 (16)
N3—Ni1—N4	78.03 (17)
O5—Ni1—N4	156.02 (15)

**Table:** 

N1—C6—C7—N2	−3.5 (6)
N3—C18—C19—N4	−0.1 (7)

**Table 2:** Hydrogen-bond geometry (Å, °)

*D*—H⋯*A*	*D*—H	H⋯*A*	*D*⋯*A*	*D*—H⋯*A*
O12—H12*B*⋯O6^i^	0.83	2.02	2.783 (6)	153
O12—H12*A*⋯O2	0.83	1.94	2.755 (5)	165
O11—H11*B*⋯O12^ii^	0.83	1.84	2.662 (5)	172
O11—H11*A*⋯O9^iii^	0.83	1.99	2.821 (6)	177
O10—H10*B*⋯O5^ii^	0.83	2.22	2.953 (5)	148
O10—H10*A*⋯O2	0.83	2.05	2.822 (6)	155
O9—H9*B*⋯O6^iii^	0.83	1.89	2.694 (5)	162
O9—H9*A*⋯O10^iv^	0.83	1.90	2.714 (5)	167
O7—H7⋯O11^v^	0.84	1.69	2.513 (5)	166
O3—H3⋯O9	0.84	1.79	2.614 (5)	166

Symmetry codes: (i) 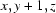; (ii) 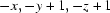; (iii) 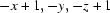; (iv) 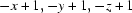; (v) 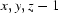.

**Fig. 1.**
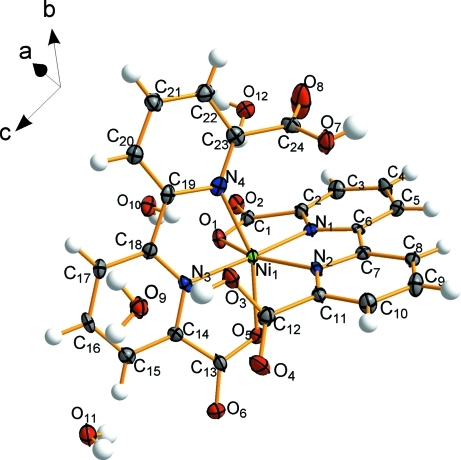
The molecule structure of the title compound, with atom labels and 50% probability displacement ellipsoids.

**Fig. 2.**
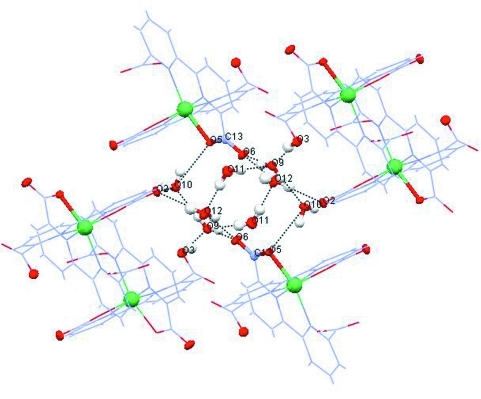
The ligand assisted hydrogen-bond cage like unit in the compound. The two complex units linked with O11 atoms and all the H atoms, have been omitted for clarity.

**Fig. 3.**
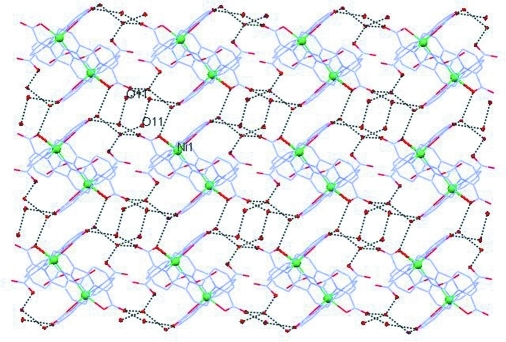
The packing of the title compound showing hydrogen bonds (dashed lines) holding the molecules together in the crystal structure. The two complex units linked with O11 atoms and all the H atoms, have been omitted for clarity.

## supplementary crystallographic information

### Comment

Pyridylcarboxylic acid is the 'ligand star' in coordination chemistry at all times (Gao *et al.*, 2006). Many multidentate ligands containing N– or O–donors, such as pyridine-2,6-dicarboxylic acid (Liu *et al.*, 2008), 2,2'-dipyridine-4,4'-dicarboxylic acid (Law *et al.*, 2007), 2,2'-dipyridine-5,5'-dicarboxylic acid (Szeto *et al.*, 2006) have been widely used because of their diverse coordination modes, which make the final architecture turn more stable and fascinating. Furthermore, they also offer more supramolecule contacts, such as hydrogen bonding or π···π stacking interactions, which further make the whole framework more stable (Robin & Fromm, 2006). However, a complex with the title ligand is still rare (Bünzli *et al.*, 2000; Knight *et al.*, 2006).

The molecule of the title complex (Fig. 1), shows that the Ni atom is located at the centre of a distorted octahedron of six coordinating atoms, four N atoms and two O atoms from the same two tridentating chelated 6-carboxy-2,2'-bipyridine-6'-carboxylate ligands L. The coordinated bipyridine fragments are nearly coplanar [torsion angles = 0.1 (7) and 3.5 (6)°, Table 1].

Three different hydrogen-bond ring patterns (see Table 2) are found in the structure: *R*_5_^3^(10) (formed by O9-H9A..O10-H10A..O2..H12A-O12-H12B.. O6..H9B-); *R*_6_^5^(14) (formed by O11-H11B..O12-H12A..O2..H10A-O10-H10B. .O5-C13-O6..H9B-O9..H11A-); and *R*_5_^5^(12) (formed by O11-H11B..O12- H12B..O6-C13-O5..H10B-O10..H9A-O9..H11A-) (Bernstein *et al.*, 1995). The intermolecular O—H···O hydrogen bonded cage-like units (assisted by the ligands L (Fig.2) and consisted of three different hydrogen-bond ring patterns), with four intermolecular hydrogen bonds, two O3—H3···O9 and two O7—H7···O11v (with H-bond patterns D, Bernstein *et al.*, 1995), link eight adjacent NiII centers to form a three-dimensional framework (Fig.3). Face-to-face π-stacking interactions between inversion related pyridine rings with Cg5···Cg6^vi^ and Cg7···Cg8^iv^ distances of 3.548 (3) and 3.662 (3) Å (perpendicular distances between the respective rings are 3.314 and 3.438 Å) make the title compound more stable. Cg5, Cg6, Cg7, and Cg8 are the centroids of the pyridine rings (N1, C2 - C6), (N2, C7 - C11), (N3, C14 - C18) and (N4, C19 - C23), respectively. Symmetry codes: (vi) = *-x, -y, -z*, and (iv) = 1 - *x*, 1 - *y*, 1 - *z*, see Fig. 1.

### Experimental

The title compound was obtained by the reaction of the mixture of Ni(NO_3_)_2_.6H_2_O, and 2,2'-dipyridine-6,6'-dicarboxylic acid in a molar ratio of 1:0.8 and 10 ml of water under hydrothermal conditions (at 413 K  for 6 days and cooled to room temperature with a 5°C h^-1^ rate). The green block crystals were washed by water, ethanol. (Yield: 68%)

### Refinement

The H atoms were placed in geometrically idealized positions (C—H = 0.95 Å and O—H = 0.82–0.84 Å), with *U*_iso_(H) = 1.2*U*_eq_(C) and *U*_iso_(H) = 1.5*U*_eq_(O).

### Crystal data

**Table:**

[Ni(C_12_H_7_N_2_O_4_)_2_]·4H_2_O	*Z* = 2
*M**_r_* = 617.17	*F*(000) = 636
Triclinic, *P*1	*D*_x_ = 1.659 Mg m^−^^3^
*a* = 9.990 (2) Å	Mo *K*α radiation, λ = 0.71073 Å
*b* = 10.896 (2) Å	Cell parameters from 3636 reflections
*c* = 12.565 (3) Å	θ = 1.8–27.9°
α = 112.97 (3)°	µ = 0.86 mm^−^^1^
β = 100.09 (3)°	*T* = 113 K
γ = 90.30 (3)°	Block, green
*V* = 1235.7 (4) Å^3^	0.20 × 0.10 × 0.08 mm

### Data collection

**Table:**

Rigaku Saturn CCD area-detector diffractometer	4305 independent reflections
Radiation source: rotating anode	2745 reflections with *I* > 2σ(*I*)
confocal	*R*_int_ = 0.126
Detector resolution: 7.31 pixels mm^-1^	θ_max_ = 25.0°, θ_min_ = 1.8°
ω and φ scans	*h* = −10→11
Absorption correction: multi-scan (*CrystalClear*; Rigaku/MSC, 2005)	*k* = −12→11
*T*_min_ = 0.846, *T*_max_ = 0.934	*l* = −14→14
7165 measured reflections	

### Refinement

**Table:**

Refinement on *F*^2^	Primary atom site location: structure-invariant direct methods
Least-squares matrix: full	Secondary atom site location: difference Fourier map
*R*[*F*^2^ > 2σ(*F*^2^)] = 0.068	Hydrogen site location: inferred from neighbouring sites
*wR*(*F*^2^) = 0.211	H-atom parameters constrained
*S* = 1.05	*w* = 1/[σ^2^(*F*_o_^2^) + (0.1025*P*)^2^] where *P* = (*F*_o_^2^ + 2*F*_c_^2^)/3
4305 reflections	(Δ/σ)_max_ < 0.001
372 parameters	Δρ_max_ = 0.96 e Å^−^^3^
0 restraints	Δρ_min_ = −0.75 e Å^−^^3^

### Special details

**Table:**

Geometry. All e.s.d.'s (except the e.s.d. in the dihedral angle between two l.s. planes) are estimated using the full covariance matrix. The cell e.s.d.'s are taken into account individually in the estimation of e.s.d.'s in distances, angles and torsion angles; correlations between e.s.d.'s in cell parameters are only used when they are defined by crystal symmetry. An approximate (isotropic) treatment of cell e.s.d.'s is used for estimating e.s.d.'s involving l.s. planes.
Refinement. Refinement of *F*^2^ against ALL reflections. The weighted *R*-factor *wR* and goodness of fit *S* are based on *F*^2^, conventional *R*-factors *R* are based on *F*, with *F* set to zero for negative *F*^2^. The threshold expression of *F*^2^ > σ(*F*^2^) is used only for calculating *R*-factors(gt) *etc*. and is not relevant to the choice of reflections for refinement. *R*-factors based on *F*^2^ are statistically about twice as large as those based on *F*, and *R*- factors based on ALL data will be even larger.

### Fractional atomic coordinates and isotropic or equivalent isotropic displacement parameters (Å^2^)

**Table:**

	*x*	*y*	*z*	*U*_iso_*/*U*_eq_	
Ni1	0.26176 (6)	0.23748 (6)	0.25373 (5)	0.0128 (3)	
O1	0.1825 (4)	0.4096 (3)	0.3635 (3)	0.0170 (9)	
O2	0.0298 (4)	0.5584 (4)	0.3582 (3)	0.0206 (9)	
O3	0.5431 (4)	0.0527 (4)	0.2154 (4)	0.0236 (10)	
H3	0.6006	0.0445	0.2692	0.035*	
O4	0.4382 (5)	−0.1285 (5)	0.2149 (4)	0.0463 (15)	
O5	0.1553 (4)	0.1112 (3)	0.3108 (3)	0.0168 (8)	
O6	0.1883 (4)	0.0088 (4)	0.4363 (4)	0.0259 (10)	
O7	0.3576 (4)	0.2726 (4)	−0.0101 (4)	0.0279 (10)	
H7	0.3161	0.2681	−0.0759	0.042*	
O8	0.2810 (5)	0.4775 (5)	0.0577 (5)	0.0513 (15)	
N1	0.1132 (4)	0.2644 (4)	0.1406 (4)	0.0130 (10)	
N2	0.2678 (4)	0.0647 (4)	0.0940 (4)	0.0113 (9)	
N3	0.3979 (4)	0.2352 (4)	0.3886 (4)	0.0130 (10)	
N4	0.4370 (4)	0.3585 (4)	0.2514 (4)	0.0142 (10)	
C1	0.0871 (5)	0.4564 (5)	0.3123 (5)	0.0161 (12)	
C2	0.0440 (5)	0.3747 (5)	0.1788 (5)	0.0149 (12)	
C3	−0.0525 (5)	0.4060 (5)	0.1044 (5)	0.0194 (13)	
H3A	−0.0980	0.4855	0.1320	0.023*	
C4	−0.0825 (5)	0.3167 (5)	−0.0151 (5)	0.0192 (13)	
H4	−0.1482	0.3361	−0.0696	0.023*	
C5	−0.0156 (5)	0.2000 (5)	−0.0527 (5)	0.0179 (13)	
H5	−0.0385	0.1370	−0.1321	0.021*	
C6	0.0848 (5)	0.1768 (5)	0.0271 (4)	0.0110 (11)	
C7	0.1705 (5)	0.0604 (5)	0.0014 (5)	0.0108 (11)	
C8	0.1510 (5)	−0.0456 (5)	−0.1094 (5)	0.0134 (12)	
H8	0.0834	−0.0443	−0.1723	0.016*	
C9	0.2307 (5)	−0.1519 (5)	−0.1265 (5)	0.0217 (13)	
H9	0.2192	−0.2246	−0.2012	0.026*	
C10	0.3280 (5)	−0.1511 (5)	−0.0330 (5)	0.0188 (13)	
H10	0.3835	−0.2237	−0.0422	0.023*	
C11	0.3433 (5)	−0.0420 (5)	0.0749 (5)	0.0141 (12)	
C12	0.4464 (5)	−0.0429 (5)	0.1771 (5)	0.0185 (13)	
C13	0.2244 (6)	0.0881 (5)	0.3940 (5)	0.0188 (13)	
C14	0.3639 (5)	0.1643 (5)	0.4469 (5)	0.0154 (12)	
C15	0.4503 (5)	0.1663 (5)	0.5468 (5)	0.0177 (12)	
H15	0.4258	0.1160	0.5885	0.021*	
C16	0.5741 (5)	0.2441 (5)	0.5845 (5)	0.0172 (12)	
H16	0.6354	0.2474	0.6527	0.021*	
C17	0.6077 (5)	0.3174 (5)	0.5216 (5)	0.0162 (12)	
H17	0.6919	0.3705	0.5461	0.019*	
C18	0.5161 (5)	0.3109 (5)	0.4234 (5)	0.0139 (12)	
C19	0.5397 (5)	0.3818 (5)	0.3460 (5)	0.0142 (12)	
C20	0.6567 (5)	0.4637 (5)	0.3709 (5)	0.0175 (12)	
H20	0.7262	0.4751	0.4369	0.021*	
C21	0.6713 (6)	0.5292 (5)	0.2978 (5)	0.0211 (13)	
H21	0.7510	0.5860	0.3130	0.025*	
C22	0.5682 (6)	0.5107 (5)	0.2029 (5)	0.0215 (13)	
H22	0.5741	0.5566	0.1531	0.026*	
C23	0.4553 (5)	0.4231 (5)	0.1817 (5)	0.0166 (12)	
C24	0.3523 (5)	0.3942 (5)	0.0712 (5)	0.0189 (12)	
O9	0.7524 (3)	0.0424 (4)	0.3682 (3)	0.0213 (9)	
H9A	0.8078	0.1092	0.3909	0.026*	
H9B	0.7826	0.0158	0.4206	0.026*	
O10	0.0754 (4)	0.7452 (4)	0.5945 (3)	0.0225 (9)	
H10A	0.0597	0.6738	0.5353	0.027*	
H10B	−0.0039	0.7536	0.6057	0.027*	
O11	0.2191 (4)	0.2207 (4)	0.7879 (4)	0.0285 (10)	
H11A	0.2309	0.1439	0.7435	0.034*	
H11B	0.1365	0.2302	0.7714	0.034*	
O12	0.0448 (4)	0.7657 (4)	0.2859 (4)	0.0331 (11)	
H12A	0.0511	0.7113	0.3179	0.040*	
H12B	0.0670	0.8384	0.3429	0.040*	

### Atomic displacement parameters (Å^2^)

**Table:**

	*U*^11^	*U*^22^	*U*^33^	*U*^12^	*U*^13^	*U*^23^
Ni1	0.0129 (4)	0.0140 (4)	0.0094 (4)	0.0003 (3)	−0.0015 (3)	0.0039 (3)
O1	0.0182 (19)	0.019 (2)	0.011 (2)	0.0034 (16)	0.0013 (16)	0.0030 (16)
O2	0.026 (2)	0.017 (2)	0.016 (2)	0.0074 (17)	0.0057 (18)	0.0026 (17)
O3	0.022 (2)	0.031 (2)	0.021 (2)	−0.0004 (18)	−0.0032 (18)	0.0160 (19)
O4	0.049 (3)	0.057 (3)	0.041 (3)	−0.021 (2)	−0.018 (3)	0.039 (3)
O5	0.0155 (18)	0.0175 (19)	0.017 (2)	−0.0024 (15)	−0.0002 (16)	0.0075 (16)
O6	0.026 (2)	0.028 (2)	0.025 (2)	−0.0090 (18)	−0.0022 (19)	0.016 (2)
O7	0.030 (2)	0.024 (2)	0.023 (2)	0.0009 (18)	−0.0092 (19)	0.0076 (19)
O8	0.056 (3)	0.030 (3)	0.047 (3)	0.015 (2)	−0.022 (3)	0.005 (2)
N1	0.012 (2)	0.016 (2)	0.012 (2)	−0.0013 (18)	0.0019 (18)	0.006 (2)
N2	0.011 (2)	0.015 (2)	0.008 (2)	0.0007 (17)	0.0021 (18)	0.0052 (18)
N3	0.016 (2)	0.014 (2)	0.009 (2)	0.0040 (18)	0.0026 (19)	0.0040 (18)
N4	0.013 (2)	0.010 (2)	0.016 (2)	0.0051 (17)	0.0036 (19)	0.0015 (19)
C1	0.019 (3)	0.013 (3)	0.013 (3)	0.000 (2)	0.001 (2)	0.003 (2)
C2	0.018 (3)	0.013 (3)	0.013 (3)	0.002 (2)	0.003 (2)	0.004 (2)
C3	0.015 (3)	0.021 (3)	0.021 (3)	0.004 (2)	0.003 (2)	0.008 (3)
C4	0.017 (3)	0.023 (3)	0.017 (3)	0.005 (2)	−0.001 (2)	0.010 (2)
C5	0.017 (3)	0.017 (3)	0.017 (3)	−0.004 (2)	−0.002 (2)	0.007 (2)
C6	0.015 (3)	0.011 (3)	0.007 (3)	−0.001 (2)	0.005 (2)	0.004 (2)
C7	0.010 (2)	0.011 (2)	0.012 (3)	0.001 (2)	0.004 (2)	0.004 (2)
C8	0.013 (3)	0.014 (3)	0.012 (3)	−0.001 (2)	0.002 (2)	0.004 (2)
C9	0.022 (3)	0.023 (3)	0.014 (3)	0.001 (2)	0.002 (3)	0.001 (2)
C10	0.021 (3)	0.018 (3)	0.016 (3)	0.006 (2)	0.002 (2)	0.005 (2)
C11	0.014 (3)	0.018 (3)	0.011 (3)	0.000 (2)	0.001 (2)	0.006 (2)
C12	0.018 (3)	0.017 (3)	0.018 (3)	0.002 (2)	0.001 (2)	0.006 (2)
C13	0.022 (3)	0.022 (3)	0.012 (3)	0.002 (2)	−0.002 (2)	0.008 (2)
C14	0.018 (3)	0.014 (3)	0.014 (3)	0.000 (2)	0.000 (2)	0.007 (2)
C15	0.020 (3)	0.018 (3)	0.015 (3)	0.003 (2)	0.003 (2)	0.007 (2)
C16	0.019 (3)	0.023 (3)	0.009 (3)	0.002 (2)	−0.002 (2)	0.007 (2)
C17	0.017 (3)	0.017 (3)	0.010 (3)	−0.001 (2)	−0.001 (2)	0.002 (2)
C18	0.013 (3)	0.011 (2)	0.015 (3)	0.002 (2)	0.000 (2)	0.004 (2)
C19	0.016 (3)	0.011 (3)	0.011 (3)	0.003 (2)	0.003 (2)	−0.001 (2)
C20	0.017 (3)	0.017 (3)	0.017 (3)	0.001 (2)	0.002 (2)	0.005 (2)
C21	0.021 (3)	0.026 (3)	0.016 (3)	0.000 (2)	0.004 (2)	0.008 (3)
C22	0.024 (3)	0.019 (3)	0.020 (3)	−0.001 (2)	0.007 (3)	0.006 (2)
C23	0.018 (3)	0.015 (3)	0.016 (3)	0.008 (2)	0.006 (2)	0.005 (2)
C24	0.018 (3)	0.022 (3)	0.020 (3)	0.004 (2)	0.005 (2)	0.011 (3)
O9	0.019 (2)	0.022 (2)	0.021 (2)	0.0013 (16)	−0.0014 (18)	0.0083 (18)
O10	0.0187 (19)	0.025 (2)	0.019 (2)	0.0007 (17)	0.0052 (18)	0.0025 (18)
O11	0.030 (2)	0.029 (2)	0.021 (2)	0.0026 (19)	−0.0017 (19)	0.0071 (19)
O12	0.042 (3)	0.027 (2)	0.028 (3)	−0.008 (2)	−0.003 (2)	0.013 (2)

### Geometric parameters (Å, °)

**Table:**

Ni1—N1	1.975 (4)	C7—C8	1.399 (7)
Ni1—N3	1.987 (4)	C8—C9	1.377 (7)
Ni1—O1	2.104 (4)	C8—H8	0.9500
Ni1—O5	2.136 (4)	C9—C10	1.385 (7)
Ni1—N2	2.161 (5)	C9—H9	0.9500
Ni1—N4	2.197 (4)	C10—C11	1.394 (7)
O1—C1	1.276 (6)	C10—H10	0.9500
O2—C1	1.231 (6)	C11—C12	1.503 (6)
O3—C12	1.299 (6)	C13—C14	1.519 (7)
O3—H3	0.8400	C14—C15	1.385 (7)
O4—C12	1.207 (6)	C15—C16	1.394 (7)
O5—C13	1.260 (6)	C15—H15	0.9500
O6—C13	1.258 (6)	C16—C17	1.399 (7)
O7—C24	1.329 (7)	C16—H16	0.9500
O7—H7	0.8400	C17—C18	1.379 (7)
O8—C24	1.202 (6)	C17—H17	0.9500
N1—C6	1.348 (7)	C18—C19	1.505 (7)
N1—C2	1.352 (6)	C19—C20	1.379 (8)
N2—C11	1.354 (6)	C20—C21	1.390 (7)
N2—C7	1.364 (6)	C20—H20	0.9500
N3—C14	1.332 (7)	C21—C22	1.380 (7)
N3—C18	1.339 (6)	C21—H21	0.9500
N4—C23	1.354 (7)	C22—C23	1.392 (7)
N4—C19	1.365 (6)	C22—H22	0.9500
C1—C2	1.539 (7)	C23—C24	1.496 (7)
C2—C3	1.363 (6)	O9—H9A	0.8341
C3—C4	1.410 (8)	O9—H9B	0.8302
C3—H3A	0.9500	O10—H10A	0.8296
C4—C5	1.392 (7)	O10—H10B	0.8283
C4—H4	0.9500	O11—H11A	0.8278
C5—C6	1.387 (6)	O11—H11B	0.8319
C5—H5	0.9500	O12—H12A	0.8320
C6—C7	1.495 (7)	O12—H12B	0.8299
			
N1—Ni1—N3	170.11 (18)	C7—C8—H8	120.3
N1—Ni1—O1	78.57 (16)	C8—C9—C10	118.9 (5)
N3—Ni1—O1	91.64 (15)	C8—C9—H9	120.5
N1—Ni1—O5	100.61 (15)	C10—C9—H9	120.5
N3—Ni1—O5	78.13 (15)	C9—C10—C11	118.9 (5)
O1—Ni1—O5	92.18 (14)	C9—C10—H10	120.5
N1—Ni1—N2	78.15 (17)	C11—C10—H10	120.5
N3—Ni1—N2	111.50 (16)	N2—C11—C10	123.5 (4)
O1—Ni1—N2	156.37 (14)	N2—C11—C12	117.6 (5)
O5—Ni1—N2	88.21 (15)	C10—C11—C12	118.8 (4)
N1—Ni1—N4	103.32 (16)	O4—C12—O3	125.4 (5)
N3—Ni1—N4	78.03 (17)	O4—C12—C11	121.3 (5)
O1—Ni1—N4	91.03 (15)	O3—C12—C11	113.3 (4)
O5—Ni1—N4	156.02 (15)	O6—C13—O5	126.1 (5)
N2—Ni1—N4	98.14 (17)	O6—C13—C14	117.9 (4)
C1—O1—Ni1	115.9 (3)	O5—C13—C14	116.0 (5)
C12—O3—H3	109.5	N3—C14—C15	120.9 (5)
C13—O5—Ni1	114.2 (3)	N3—C14—C13	113.2 (4)
C24—O7—H7	109.5	C15—C14—C13	125.8 (5)
C6—N1—C2	121.0 (4)	C14—C15—C16	118.3 (5)
C6—N1—Ni1	120.6 (3)	C14—C15—H15	120.9
C2—N1—Ni1	118.4 (3)	C16—C15—H15	120.9
C11—N2—C7	116.5 (5)	C15—C16—C17	119.7 (5)
C11—N2—Ni1	130.6 (3)	C15—C16—H16	120.1
C7—N2—Ni1	112.6 (3)	C17—C16—H16	120.1
C14—N3—C18	121.8 (4)	C18—C17—C16	118.6 (5)
C14—N3—Ni1	118.1 (3)	C18—C17—H17	120.7
C18—N3—Ni1	119.9 (3)	C16—C17—H17	120.7
C23—N4—C19	115.6 (4)	N3—C18—C17	120.6 (5)
C23—N4—Ni1	132.1 (3)	N3—C18—C19	114.9 (4)
C19—N4—Ni1	111.9 (3)	C17—C18—C19	124.5 (5)
O2—C1—O1	127.0 (5)	N4—C19—C20	123.8 (5)
O2—C1—C2	118.6 (4)	N4—C19—C18	114.7 (4)
O1—C1—C2	114.4 (5)	C20—C19—C18	121.5 (4)
N1—C2—C3	122.0 (5)	C19—C20—C21	118.9 (5)
N1—C2—C1	112.4 (4)	C19—C20—H20	120.6
C3—C2—C1	125.6 (5)	C21—C20—H20	120.6
C2—C3—C4	117.9 (5)	C22—C21—C20	119.0 (5)
C2—C3—H3A	121.1	C22—C21—H21	120.5
C4—C3—H3A	121.1	C20—C21—H21	120.5
C5—C4—C3	119.8 (5)	C21—C22—C23	118.5 (5)
C5—C4—H4	120.1	C21—C22—H22	120.7
C3—C4—H4	120.1	C23—C22—H22	120.7
C6—C5—C4	119.2 (5)	N4—C23—C22	124.1 (5)
C6—C5—H5	120.4	N4—C23—C24	118.6 (5)
C4—C5—H5	120.4	C22—C23—C24	117.2 (5)
N1—C6—C5	120.0 (5)	O8—C24—O7	125.3 (5)
N1—C6—C7	113.5 (4)	O8—C24—C23	122.1 (5)
C5—C6—C7	126.5 (5)	O7—C24—C23	112.4 (4)
N2—C7—C8	122.7 (4)	H9A—O9—H9B	97.5
N2—C7—C6	115.1 (4)	H10A—O10—H10B	97.1
C8—C7—C6	122.2 (4)	H11A—O11—H11B	106.3
C9—C8—C7	119.4 (5)	H12A—O12—H12B	102.5
C9—C8—H8	120.3		
			
N1—Ni1—O1—C1	−3.7 (4)	Ni1—N1—C6—C7	2.8 (6)
N3—Ni1—O1—C1	177.7 (4)	C4—C5—C6—N1	2.4 (8)
O5—Ni1—O1—C1	−104.1 (4)	C4—C5—C6—C7	−177.5 (5)
N2—Ni1—O1—C1	−13.6 (6)	C11—N2—C7—C8	−2.2 (7)
N4—Ni1—O1—C1	99.7 (4)	Ni1—N2—C7—C8	−176.9 (4)
N1—Ni1—O5—C13	−173.4 (4)	C11—N2—C7—C6	177.3 (4)
N3—Ni1—O5—C13	−3.4 (4)	Ni1—N2—C7—C6	2.6 (5)
O1—Ni1—O5—C13	−94.6 (4)	N1—C6—C7—N2	−3.5 (6)
N2—Ni1—O5—C13	109.0 (4)	C5—C6—C7—N2	176.4 (5)
N4—Ni1—O5—C13	2.8 (6)	N1—C6—C7—C8	176.0 (4)
N3—Ni1—N1—C6	−168.7 (8)	C5—C6—C7—C8	−4.1 (8)
O1—Ni1—N1—C6	−177.2 (4)	N2—C7—C8—C9	1.3 (8)
O5—Ni1—N1—C6	−87.1 (4)	C6—C7—C8—C9	−178.1 (5)
N2—Ni1—N1—C6	−1.2 (4)	C7—C8—C9—C10	0.2 (8)
N4—Ni1—N1—C6	94.5 (4)	C8—C9—C10—C11	−0.8 (8)
N3—Ni1—N1—C2	13.4 (12)	C7—N2—C11—C10	1.5 (7)
O1—Ni1—N1—C2	5.0 (4)	Ni1—N2—C11—C10	175.2 (4)
O5—Ni1—N1—C2	95.1 (4)	C7—N2—C11—C12	−177.0 (4)
N2—Ni1—N1—C2	−179.0 (4)	Ni1—N2—C11—C12	−3.3 (7)
N4—Ni1—N1—C2	−83.3 (4)	C9—C10—C11—N2	−0.1 (8)
N1—Ni1—N2—C11	−174.7 (5)	C9—C10—C11—C12	178.4 (5)
N3—Ni1—N2—C11	3.0 (5)	N2—C11—C12—O4	117.9 (6)
O1—Ni1—N2—C11	−164.9 (4)	C10—C11—C12—O4	−60.7 (8)
O5—Ni1—N2—C11	−73.5 (4)	N2—C11—C12—O3	−64.1 (7)
N4—Ni1—N2—C11	83.3 (4)	C10—C11—C12—O3	117.3 (6)
N1—Ni1—N2—C7	−0.9 (3)	Ni1—O5—C13—O6	−174.0 (5)
N3—Ni1—N2—C7	176.8 (3)	Ni1—O5—C13—C14	6.1 (6)
O1—Ni1—N2—C7	9.0 (6)	C18—N3—C14—C15	0.1 (8)
O5—Ni1—N2—C7	100.3 (3)	Ni1—N3—C14—C15	−175.3 (4)
N4—Ni1—N2—C7	−102.9 (3)	C18—N3—C14—C13	178.8 (5)
N1—Ni1—N3—C14	83.3 (10)	Ni1—N3—C14—C13	3.3 (6)
O1—Ni1—N3—C14	91.6 (4)	O6—C13—C14—N3	173.7 (5)
O5—Ni1—N3—C14	−0.3 (4)	O5—C13—C14—N3	−6.4 (7)
N2—Ni1—N3—C14	−83.6 (4)	O6—C13—C14—C15	−7.7 (9)
N4—Ni1—N3—C14	−177.7 (4)	O5—C13—C14—C15	172.2 (5)
N1—Ni1—N3—C18	−92.2 (10)	N3—C14—C15—C16	−0.3 (8)
O1—Ni1—N3—C18	−84.0 (4)	C13—C14—C15—C16	−178.8 (5)
O5—Ni1—N3—C18	−175.9 (4)	C14—C15—C16—C17	0.1 (8)
N2—Ni1—N3—C18	100.9 (4)	C15—C16—C17—C18	0.3 (8)
N4—Ni1—N3—C18	6.7 (4)	C14—N3—C18—C17	0.3 (8)
N1—Ni1—N4—C23	−8.8 (5)	Ni1—N3—C18—C17	175.6 (4)
N3—Ni1—N4—C23	−178.8 (5)	C14—N3—C18—C19	178.7 (5)
O1—Ni1—N4—C23	−87.3 (5)	Ni1—N3—C18—C19	−6.0 (6)
O5—Ni1—N4—C23	175.0 (4)	C16—C17—C18—N3	−0.4 (8)
N2—Ni1—N4—C23	70.9 (5)	C16—C17—C18—C19	−178.7 (5)
N1—Ni1—N4—C19	163.7 (3)	C23—N4—C19—C20	−0.9 (8)
N3—Ni1—N4—C19	−6.3 (3)	Ni1—N4—C19—C20	−174.7 (4)
O1—Ni1—N4—C19	85.2 (3)	C23—N4—C19—C18	179.0 (4)
O5—Ni1—N4—C19	−12.5 (6)	Ni1—N4—C19—C18	5.2 (5)
N2—Ni1—N4—C19	−116.7 (3)	N3—C18—C19—N4	−0.1 (7)
Ni1—O1—C1—O2	−177.0 (5)	C17—C18—C19—N4	178.2 (5)
Ni1—O1—C1—C2	1.9 (6)	N3—C18—C19—C20	179.8 (5)
C6—N1—C2—C3	−3.1 (8)	C17—C18—C19—C20	−1.9 (8)
Ni1—N1—C2—C3	174.7 (4)	N4—C19—C20—C21	1.5 (8)
C6—N1—C2—C1	176.8 (4)	C18—C19—C20—C21	−178.4 (5)
Ni1—N1—C2—C1	−5.4 (6)	C19—C20—C21—C22	0.2 (8)
O2—C1—C2—N1	−178.9 (5)	C20—C21—C22—C23	−2.2 (8)
O1—C1—C2—N1	2.1 (7)	C19—N4—C23—C22	−1.3 (8)
O2—C1—C2—C3	1.0 (8)	Ni1—N4—C23—C22	170.9 (4)
O1—C1—C2—C3	−178.1 (5)	C19—N4—C23—C24	175.3 (4)
N1—C2—C3—C4	2.2 (8)	Ni1—N4—C23—C24	−12.4 (7)
C1—C2—C3—C4	−177.7 (5)	C21—C22—C23—N4	2.9 (9)
C2—C3—C4—C5	1.0 (8)	C21—C22—C23—C24	−173.8 (5)
C3—C4—C5—C6	−3.2 (8)	N4—C23—C24—O8	114.4 (7)
C2—N1—C6—C5	0.7 (7)	C22—C23—C24—O8	−68.8 (8)
Ni1—N1—C6—C5	−177.1 (4)	N4—C23—C24—O7	−70.5 (7)
C2—N1—C6—C7	−179.4 (4)	C22—C23—C24—O7	106.4 (6)

### Hydrogen-bond geometry (Å, °)

**Table:**

*D*—H···*A*	*D*—H	H···*A*	*D*···*A*	*D*—H···*A*
O12—H12B···O6^i^	0.83	2.02	2.783 (6)	153
O12—H12A···O2	0.83	1.94	2.755 (5)	165
O11—H11B···O12^ii^	0.83	1.84	2.662 (5)	172
O11—H11A···O9^iii^	0.83	1.99	2.821 (6)	177
O10—H10B···O5^ii^	0.83	2.22	2.953 (5)	148
O10—H10A···O2	0.83	2.05	2.822 (6)	155
O9—H9B···O6^iii^	0.83	1.89	2.694 (5)	162
O9—H9A···O10^iv^	0.83	1.90	2.714 (5)	167
O7—H7···O11^v^	0.84	1.69	2.513 (5)	166
O3—H3···O9	0.84	1.79	2.614 (5)	166

Symmetry codes: (i) *x*, *y*+1, *z*; (ii) −*x*, −*y*+1, −*z*+1; (iii) −*x*+1, −*y*, −*z*+1; (iv) −*x*+1, −*y*+1, −*z*+1; (v) *x*, *y*, *z*−1.
